# Kurtosis fractional anisotropy, its contrast and estimation by proxy

**DOI:** 10.1038/srep23999

**Published:** 2016-04-04

**Authors:** Brian Hansen, Sune Nørhøj Jespersen

**Affiliations:** 1 Center of Functionally Integrative Neuroscience (CFIN), Clinical Institute, Aarhus University, Aarhus, Denmark; 2 Department of Physics and Astronomy, Aarhus University, Aarhus, Denmark

## Abstract

The diffusion kurtosis observed with diffusion magnetic resonance imaging (dMRI) may vary with direction. This directional variation is summarized in the scalar kurtosis fractional anisotropy (KFA). Recent studies suggest that kurtosis anisotropy offers microstructural contrast not contained in other commonly used dMRI markers. We compare KFA to other dMRI contrasts in fixed rat brain and in human brain. We then investigate the observed contrast differences using data obtained in a physical phantom and simulations based on data from the phantom, rat spinal cord, and human brain. Lastly, we assess a strategy for rapid estimation of a computationally modest KFA proxy by evaluating its correlation to true KFA for varying number of sampling directions and signal-to-noise ratio (SNR) levels. We also map this proxy’s b-value dependency. We find that KFA supplements the contrast of other dMRI metrics – particularly fractional anisotropy (FA) which vanishes in near orthogonal fiber arrangements where KFA does not. Simulations and phantom data support this interpretation. KFA therefore supplements FA and could be useful for evaluation of complex tissue arrangements. The KFA proxy is strongly correlated to true KFA when sampling is performed along at least nine directions and SNR is high.

## Introduction

Diffusion weighted magnetic resonance imaging (dMRI) offers unrivalled sensitivity to tissue microstructure making it an important investigative and diagnostic tool. Traditional dMRI analysis has employed the diffusion tensor model in which diffusion is assumed to be Gaussian; tissue microstructure, however, causes the spin probability distribution to deviate from normal. This deviation is therefore seen as an indirect microstructural marker. The diffusion kurtosis imaging (DKI) framework^1^ captures the deviation from Gaussianity and is an increasingly popular method to further the sensitivity of dMRI to microstructure. As with diffusivity the kurtosis may be directionally dependent. The anisotropy of the diffusion tensor^2^,^3^ is described compactly by the scalar fractional anisotropy, FA^4^. Various metrics of kurtosis anisotropy have been suggested^5^,^6^ but they do not provide information of the anisotropy of the kurtosis tensor alone as they include information from the diffusion tensor in the metric. So far, only few studies (e.g.^7^,^8^) have employed them, leaving the value of these kurtosis anisotropy metrics largely unexplored. A recently^9^,^10^ proposed measure of kurtosis anisotropy (the kurtosis fractional anisotropy or KFA) is mathematically analogous to FA. This provides coherent definitions of the basic anisotropy markers derived from DTI and DKI so that KFA solely reflects the anisotropy of the kurtosis tensor without contributions from the diffusion tensor. KFA was studied extensively in^11^ using data from human brain and simulations to explore its contrast and compare KFA to previous kurtosis anisotropy metrics and the generalized fractional anisotropy^12^ calculated from an approximation of the diffusion orientation distribution function derived from DKI^13^,^14^. The scope of the present paper is limited to KFA and comparing its contrast to the contrast content of traditional dMRI metrics - including FA. Our comparison is based on data from fixed rat brain and *in vivo* human brain. We also perform an investigation of the observed contrast differences between FA and KFA using data obtained in a physical phantom and simulations based on data from the phantom, rat spinal cord, and human brain. The findings supplement and are consistent with the report in^11^. Lastly, we propose and evaluate a KFA proxy which has the advantage that it may be evaluated based on much less data than the true KFA and reconstructed rapidly without fitting. This proxy method could be suitable for the clinical setting where the time required to obtain true KFA from the kurtosis tensor may be prohibitive. We assess the KFA proxy by evaluating its correlation to true KFA for varying number of sampling directions and SNR levels. We also map the b-value dependency of the correlation between the proxy and KFA.

### Theory

In DKI the logarithm of the diffusion MRI signal 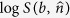 is described by the Taylor expansion^1^:


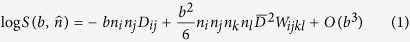


where b is the diffusion weighting applied along 
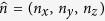
 and subscripts label Cartesian components (e.g. *i* = *x, y, z*). In our notation, summation over repeated indices is implied so that e.g. 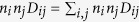. Here, the kurtosis tensor W is defined in terms of spin displacement moments as in^1^. Fitting Eq. (1) to appropriate diffusion MRI data provides several metrics obtainable from the diffusion tensor D and the kurtosis tensor W, foremost the mean diffusivity 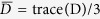, the mean kurtosis MK^15^, and the mean of the kurtosis tensor 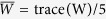
10,16. Both mean kurtosis metrics have been shown to have diagnostic value e.g.^17^,^18^,^19^; see also more complete literature surveys in^10^,^20^. The diffusion tensor D allows assessment of the tissue anisotropy through the fractional anisotropy (FA) as^4^:


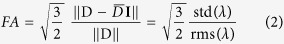


where λ are the eigenvalues of the diffusion tensor, std denotes the standard deviation, and rms the root-mean-square value. In complete analogy a kurtosis fractional anisotropy (KFA) has been introduced based on the kurtosis tensor W^9^,^10^:


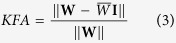


Here, as well as in Eq. (2), double vertical bars ||**A**||^2^ = <**A**, **A**> signifies the Frobenius norm of the tensor. Drawing inspiration from the rightmost expression in Eq. (2) we propose that a useful approximation of KFA might be obtained from the directional variation of the apparent kurtosis 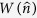 as:


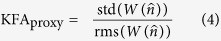


While conceptually reflecting the same information, the KFA proxy is not directly equivalent to the KFA defined in Eq. (3). Complete agreement between the two is therefore not expected, but the proxy might be useful in that it can be estimated without knowledge of the full tensor W as required in Eq. (3). The proxy definition is similar in form to the right-most expression in Eq. (2). However, no appropriate normalization factor is known for the KFA proxy and so the range of KFA and the KFA proxy cannot be expected to coincide either. It is not evident if this strategy would work and how many directions would be needed. However, a rapid estimation technique would perhaps facilitate exploration of the usefulness of KFA as a biomarker. The feasibility of the proxy strategy and its experimental dependency on number of sampled directions 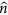, choice of b-values and signal-to-noise ratio (SNR) is the subject of the final part of this study.

## Results

**Figure 1:**
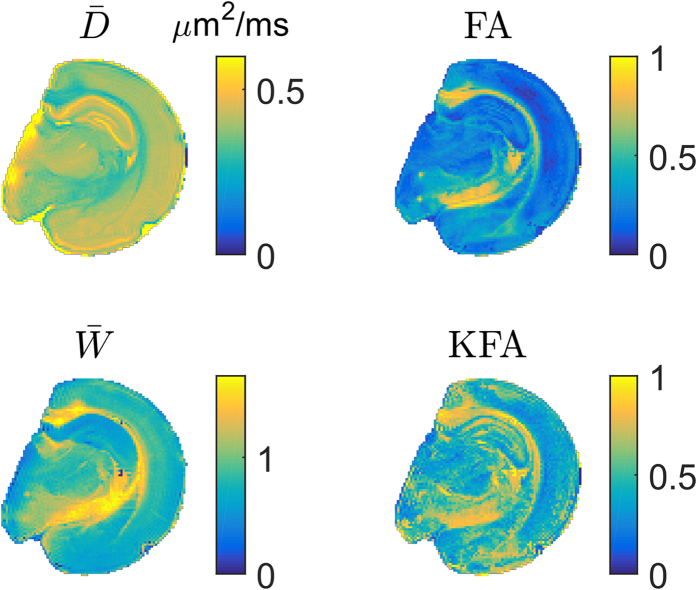
Maps of 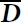, FA, 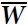, and KFA from a high resolution data set collected in fixed rat brain allows qualitative comparison of the contrast in provided by each metric.

Examples of 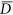, FA, 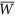, and KFA are provided in Fig. 1. Generally, the KFA is high where FA is high but KFA is also seen to possess high values in areas where FA is low e.g. in regions of the hippocampus and some areas of cortex.

**Figure 2:**
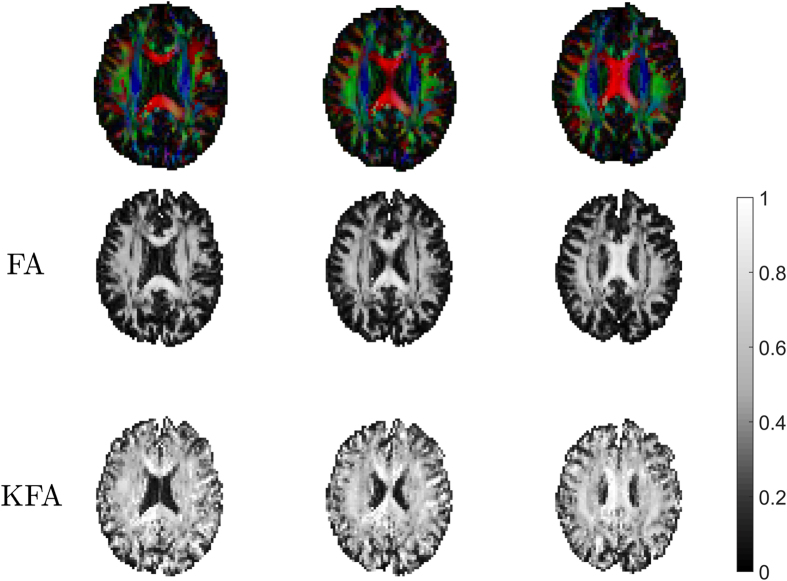
FA and KFA in normal human brain. The top row shows the FA map with fiber direction encoded in the red-green-blue color scheme (red: left-right, green: up-down, blue: in-out of plane). A low intensity band is seen in both versions of the FA maps inside the WM (e.g. as pointed out be the red arrows). The band is present in all three consecutive slices shown. KFA in the same slices does not show this behavior.

This behavior is even more apparent in the data example from human brain shown in Fig. 2. Here direction encoded color FA map^21^ (top row), FA and KFA are shown in three consecutive axial slices. In both types of FA maps, a dark band is seen to go through the white matter (WM) region in a anterior-posterior direction (indicated in one side by the arrows but seen in both hemispheres). The band is present in all three slices and is clearly inside anatomical WM, but the fiber arrangement in this bundle causes FA to vanish. In contrast, KFA only decreases slightly in these regions and is high in all WM areas but low in gray matter (GM) regions although not as low as FA. To further explore the information difference between KFA and typical dMRI metrics we list the linear correlation coefficients between KFA and 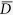, FA, and 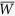 for the data in Figs 1 and 2 in Table 1. Segmentation was performed based on FA as described in the methods. Generally the correlations are weak except for the correlation between FA and as expected from visual inspection of the maps in Figs 1 and 2. However, even here the correlations are weak (0.5–0.6) most likely due to the regions where FA vanishes and KFA does not.

**Figure 3:**
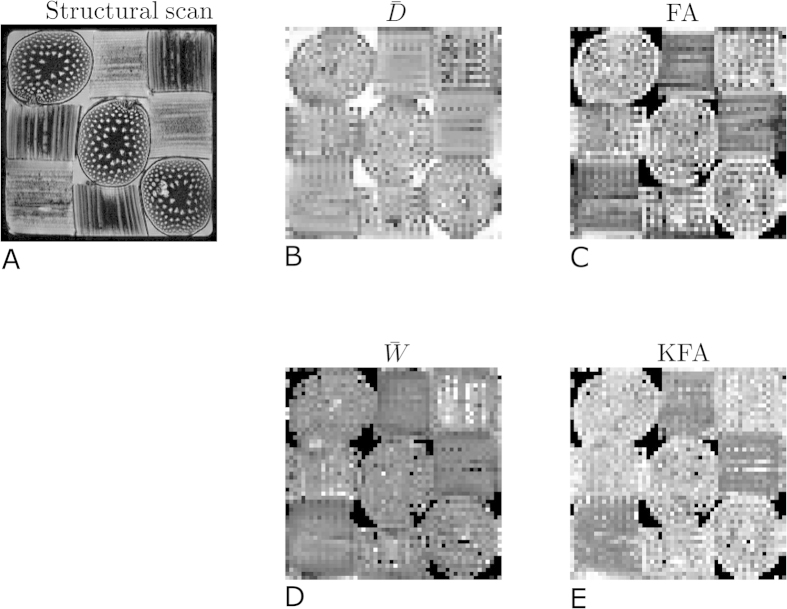
The physical phantom employed contains three orthogonal fiber directions in a 3 × 3 arrangement as seen in the structural scan. Maps of 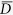, FA, 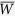, and KFA are shown in the remaining panels. Water surrounds the fibers explaining the high intensity background in the 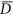 map. These regions were used to segment out the background for the further analysis.

In the following, KFAs robustness to such fiber arrangements where FA ceases to provide information is explored. Firstly, we do this using a physical phantom (asparagus) with three orthogonal fiber directions present in equal proportions. Figure 3A shows a structural scan of the phantom clearly showing each fiber bundle in the 3 × 3 arrangement and each bundle’s fiber orientation. The remaining panels show parameter maps obtained from a voxel-wise fit of Eq. (1) to DKI data collected in the phantom.

**Figure 4:**
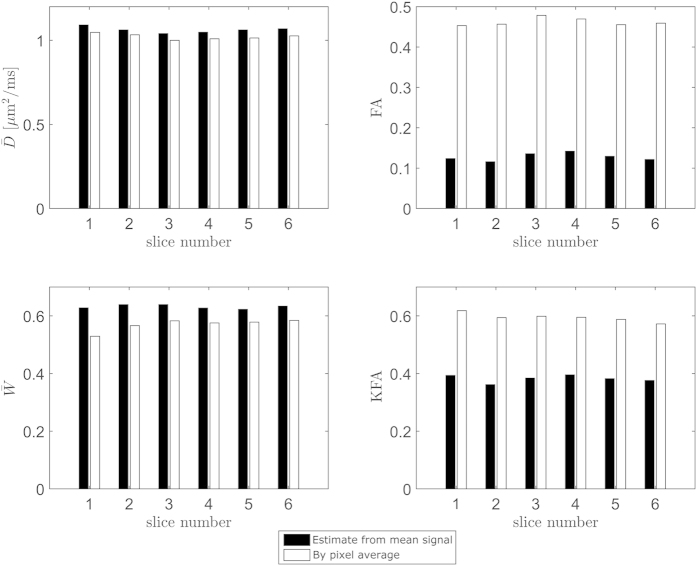
Comparison of the robustness of 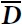, FA, 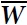, and KFA to fiber orientation heterogeneity across six image planes of the physical phantom. In each panel white bars correspond to the true parameter average. The black bars show the parameter values obtained when the DKI data from each diffusion encoding is averaged across voxels prior to fitting. This mimics the situation where low imaging resolution causes a voxel to have substantial fiber direction heterogeneity. The effect is seen to vary between parameters but clearly FA is most strongly affected and KFA much less so. In contrast, 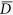 and 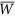 are almost unaffected.

For the analysis, we used the map of 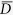 from each image plane to segment out the water-containing background voxels. When the parameter maps of 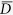, FA, 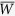, and KFA are averaged over this mask we obtain a true average of each parameter in each image plane. If instead we average the DKI data (again using the mask based on 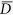) for each diffusion encoding prior to fitting with Eq. (1) we obtain values corresponding to estimation based on low resolution scans with substantial fiber orientation heterogeneity below voxel level (mimicking a 3D orthogonal fiber arrangement where FA is zero). These estimates are shown alongside the true mean values in Fig. 4. The behavior is consistent across all image slices: 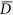 is unaffected while the 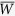 estimate obtained from voxel averaging prior to fitting is slightly higher than its true mean value. As expected for single fiber orientations both the average true FA and KFA are high while FA almost vanishes when each diffusion encoding is averaged over voxels. In this case KFA also decreases compared to the true average but to a much smaller extent than FA.

The analysis based on the physical phantom demonstrates the sensitivity of FA to the presence of multiple fiber orientations and also the relative insensitivity of KFA to these conditions. 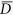 and 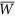 are almost immune to the fiber orientation heterogeneity. Theoretically, 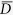 is expected to remain constant due to linearity. Apparently, averaging the signal over fiber directions causes the signal to appear more non-Gaussian (perhaps due to increased diffusion heterogeneity from averaging over compartments) reflected in the slight increase in 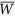. KFA on the other hand decreases slightly when the signal is averaged across directions possible due to smaller directional dependence in the averaged signal compared to a single fiber. As expected then, FA is the only parameter to show inability to accommodate these complex diffusion profiles. To further explore this behavior we performed a series of simulations based on data from the physical phantom, high resolution DKI data from rat spinal cord and human brain.

**Figure 5:**
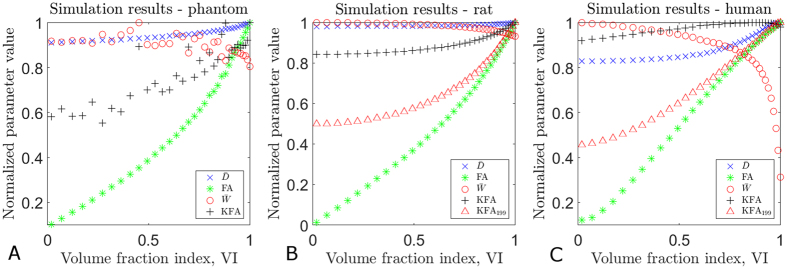
Simulations of the effect of increasing fiber orientation heterogeneity based on data from the physical phantom (**A**), fixed rat spinal cord (**B**), and WM in human brain (**C**). The curve labeled KFA_199_ shows the behavior of the KFA proxy based on an estimation from 9 directions. Overall, the observed effects are the same although effect size varies: 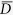 and 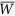 are unaffected by the increasing heterogeneity, contrarily KFA is slightly sensitive to the fiber orientation mixture but not as severely as FA which is seen to vanish almost completely when the fibers directions are present in equal proportions. Each parameter is normalized by its maximum value.

Figure 5 shows the results of simulations investigating KFA behavior with increasing tissue complexity. The simulated tissue composition is described by the volume fraction index VI which is 1 when only a single fiber direction is present (volume fraction 1) and decreases to zero when three orthogonal fiber directions exist in equal amounts (each with volume fraction 1/3). The VI is defined in the methods section where its behavior is also illustrated. In the simulations we also evaluate the behavior of other diffusion metrics as fiber orientation heterogeneity increases. The figure shows results of simulations based on the data from the physical phantom (panel A), DKI data of rat spinal cord (panel B), and normal human brain (panel C). Details on the regions/voxels used in the simulations are provided in the methods section. The simulations show the behavior of 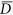, FA, 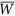, and KFA with increasing tissue complexity. In the graphs each parameter is normalized by its maximum value in the simulation. Simulation results are seen to yield similar results for all three systems: for a single orientation (VI = 1) both FA and KFA are high but FA is seen to decrease with increasing tissue complexity whereas KFA is less affected and does not vanish even for equal fiber volume fraction 1/3 (VI = 0) where FA vanishes. The simulations also show 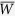 to increase slightly with increasing complexity for simulations based on preclinical data (panels 5A–B). In the simulations based on human data (panel 5C) the increase in 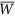 is more pronounced. We attribute this behavior to the different experimental conditions under which the data for the simulations were acquired: plant stalks/fixed tissue and strong gradients (5A–B), and *in vivo* human brain with weaker gradients (affecting DWI encoding timings). We note, however, that the overall behavior is the same. 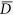 is unaffected by the mixture level. While overall behavior is the same in the three systems the simulations based on phantom data (Fig. 5A) are seen to be noisier than the other two simulations (Fig. 5B,C). This is because the phantom simulations rely on measured signals to a much higher degree than the other two simulations. This introduces more noise into the simulations which naturally affects the non-Gaussian metrics 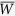 and KFA much more than the diffusion tensor derived metrics 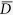 and FA.

### Fast estimation of KFA

Eq. (4) provides a strategy for obtaining a KFA proxy that can be obtained without the need for data fitting. However, the relation between this proxy and KFA is not direct and therefore we investigate how strongly the proxy correlates with KFA and how the correlation depends on acquisition details such as number of sampling directions, choice of b-values and signal-to-noise (SNR) levels.

**Figure 6:**
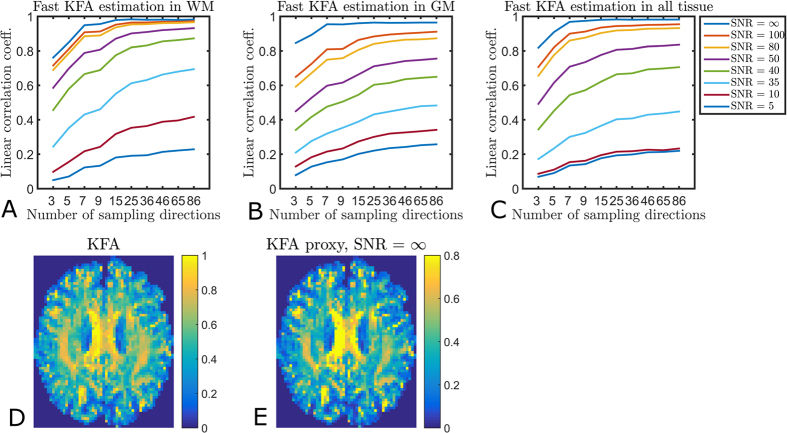
Correlation strength between KFA and the KFA proxy for a range of sampling directions and SNR levels at b = 0. Panel (**A**) shows the correlation for WM only, (**B**) shows GM behavior, and panel (**C**) shows the behavior for all tissue voxels. The bottom row compares KFA (**D**) to the idealized situation where the proxy is estimated from 86 sampling directions at infinite SNR (**E**). The proxy is not equivalent to true KFA and therefore the maps are shown on different scales. This, however, does not affect contrast offered by the proxy, which is seen to be quite similar. The similarity is also reflected in the linear correlation coefficient of 0.97 between the two maps.

Firstly, Fig. 6 shows the correlation strength between true KFA and the KFA proxy assessed using a range of sampling directions and SNR levels. In the figure, results from separate investigations of WM, GM and all tissue combined are shown (see Methods for details on segmentation). The overall behavior is the same with low correlation between KFA and the proxy at SNR levels below 50 in all three cases. Almost identical performance is seen for SNR levels 80 and 100 with correlation strengths consistently above 0.8 when the number of sampled directions ≥9. Correlation is best in WM, where a proxy estimation based on 25 directions at SNR = 80 performs as well as the ideal case of infinite SNR. For the case where both tissue types are present (Fig. 6C), the same behavior is seen.

To further assess the proxy’s correlation to true KFA the bottom row of Fig. 6 shows KFA (Fig. 6D) alongside the idealized situation where simulations are used to estimate the proxy from 86 sampling directions at infinite SNR (Fig. 6E). As expected the obtained value ranges are not identical since the proxy is not equivalent to true KFA. The maps shown in Fig. 6D,E span nearly the same range of values (0.05–1 for KFA and 0.03–1 for the proxy) with nearly identical standard deviations of 0.23 for KFA and 0.22 for the proxy. However, comparison of the histograms of these two maps (data not shown) reveals a pile up of low to intermediate (0.2–0.4) values in the proxy causing a lower mean value for the proxy of 0.36 compared to 0.49 for true KFA. The proxy is seen to assume lower values than true KFA in the same WM regions where FA assumes low values (the dark bands) in Fig. 2. This behavior is predicted by the simulations (Fig. 5B,C), where the proxy estimated from nine directions (KFA_199_) is seen to decrease more than true KFA for low VI. However, even here the FKA proxy still indicates higher diffusion anisotropy than FA in configurations where diffusion anisotropy is expected. For these reasons we show the maps on different scales to expose similarities and differences. A common way of comparing contrast in images is by way of the root-mean-square (RMS) contrast^22^. Normalizing both maps to the range 0–1 we obtain very similar RMS contrasts for the two maps: 0.24 for KFA and 0.22 for the proxy. Overall, the contrast offered by the proxy is seen to be quite similar to true KFA and the linear correlation coefficient between the two maps in Fig. 6D,E is 0.97.

**Figure 7:**
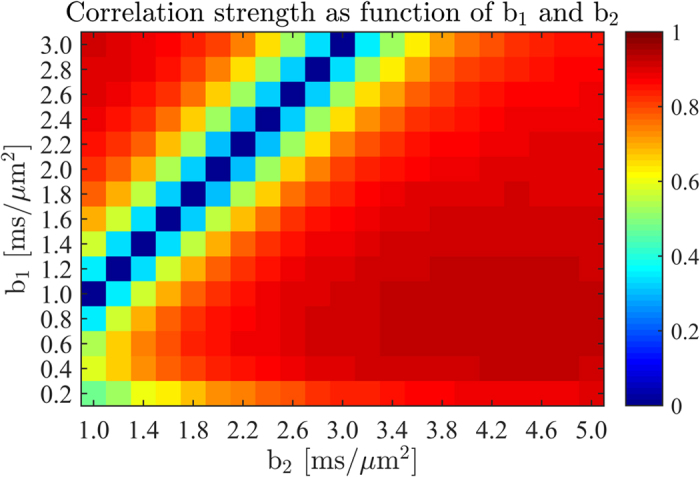
Linear correlation coefficient between KFA and the KFA proxy evaluated from nine encoding directions as function of b-values for the two shells b_1_ and b_2_.

**Figure 8:**
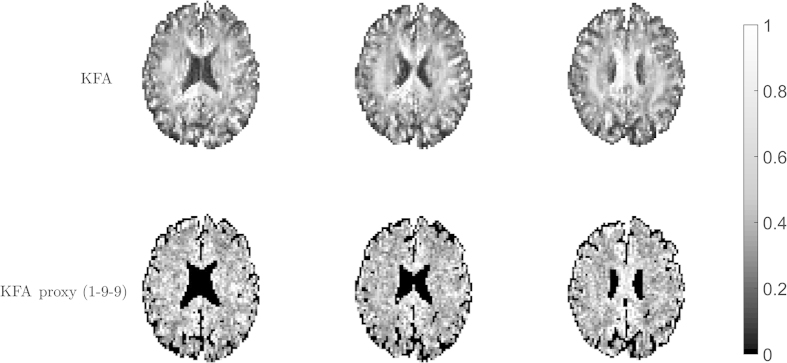
Comparison of true KFA to a proxy acquisition using 9 directions, b_1_ = 1 ms/μm^2^ and b_2_ = 2.6 ms/μm^2^ at SNR at b = 0 just below 40 in the same slices as shown in Fig. 2. CSF has been segmented out in the proxy maps. The linear correlation coefficients for the three slices are (left to right): 0.55, 0.54, and 0.56 in agreement with the simulations.

**Figure 9:**
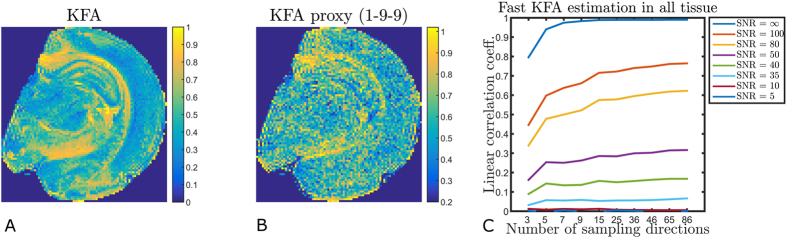
Comparison of true KFA (**A**) to a proxy acquisition (**B**) using 9 directions, b_1_ = 1 ms/μm^2^ and b_2_ = 2.6 ms/μm^2^ in rat brain at an SNR at b = 0 of approximately 75 in the same slice as shown in Fig. 1. The linear correlation coefficient between these maps is 0.49 in agreement with simulations of proxy estimation efficiency in fixed tissue (**C**). Still, some contrast similarity remains, particularly in areas with high KFA.

Having thus established the correlation between KFA and the KFA proxy as well as the proxy’s requirements in terms of SNR and encoding directions we turn to the choice of b-values (b_1_ and b_2_) of the two shells required for proxy estimation. Figure 7 shows the linear correlation coefficient as a function of b_1_ and b_2_ obtained from simulation (simulation SNR = 100 at b = 0, 9 encoding directions, all tissue types included). We note that b_1_ = b_2_ is not a valid combination which explains the estimation behavior in the vicinity of the b_1_ = b_2_ line in the map. From the map we see that choosing a large b_2_-value (b_1_ ~ 1 ms/μm^2^, b_2 _~ 5 ms/μm^2^) produces a proxy with a correlation to true KFA of ~0.93. However, this is well outside the b-value range typically employed in DKI due to the non-negligible influence of higher order effects at b > 3 ms/μm^2^ 15. The b-value choice recommended for fast kurtosis imaging in^20^ (b_1_ = 1 ms/μm^2^ and b_2_ = 2.4–2.6 ms/μm^2^) provides a correlation of ~0.9, meaning that a good KFA estimate can be obtained from the scheme in^20^ given sufficiently high SNR. Using 25 directions the correlation increases slightly to 0.93 for these b-values and peaks at 0.96 for b_1_ = 1 ms/μm^2^, b_2_ = ~5 ms/μm^2^. For SNR = 60 (at b = 0) these same b-value combinations provide estimates with correlations of 0.80 (nine directions) and 0.85 (25 directions) respectively. To illustrate the method performance at a more standard SNR level Fig. 8 shows a realization of the proxy acquisition using 9 directions, b_1_ = 1 ms/μm^2^ and b_2_ = 2.6 ms/μm^2^ which had SNR at b = 0 just below 40. The data for this was acquired as part of the human acquisition that produced Fig. 2 and the same slices are shown. CSF has been segmented out in the proxy maps to ease comparison since estimation in these regions are dominated by the low SNR at b_2_. The linear correlation coefficients between KFA and the proxy for the three slices shown are (left to right): 0.55, 0.54, and 0.56 in agreement with the simulations in Fig. 6C. Figure 9 shows a realization of the proxy acquisition in fixed rat brain also using 9 directions (b_1_ = 1 ms/μm^2^ and b_2_ = 2.6 ms/μm^2^). The linear correlation between the maps in Fig. 9A,B is 0.49. In this data set SNR at b = 0 was approximately 75 and so the performance is seen to be in agreement with the simulations based on the fixed rat brain data in Fig. 9C. This indicates different b-value and SNR requirements for the proxy scheme in fixed tissue and *in vivo*. A b-value optimization similar to Fig. 7 for the nine direction KFA proxy in fixed rat brain provides optimal b-values at b_1_ = 1.6 ms/μm^2^ and b_2_ = 5.0 ms/μm^2^ providing a correlation of 0.83.

## Discussion

Enhancing dMRI’s sensitivity to microstructure and providing an ability to distinguish various microstructural compositions of tissue is important for improved diagnostics. Many promising dMRI strategies employ nonstandard spin preparations^23^,^24^,^25^,^26^ to reduce scan time and provide alternative diffusion contrasts. However, these techniques may not be available to most users and they may not be straightforward to implement on clinical systems. The KFA is a fruitful alternative because it may be estimated from DKI acquisitions using standard dMRI sequences and offers contrast in areas where the tissue’s fiber composition complexity causes FA to fail as a reporter of tissue anisotropy. Establishing a direct biological interpretation of KFA presents an interesting challenge. Studies comparing MR microscopy directly to tissue histology as done for DTI in^27^,^28^,^29^ might serve as inspiration for such efforts.

After its original proposal in^9^,^10^ KFA was studied extensively in^11^ where its use was motivated and some features of KFA were demonstrated by comparison to FA using human data and simulations. The contrast differences between KFA and other diffusion maps noted here in rat hippocampus (Fig. 1) were also observed in the deep brain structures lenticular nucleus and thalamus in human brain in^11^. That study also demonstrated KFA’s robustness to fiber configurations where FA vanishes despite a high degree of angular dependence in the diffusion profile. Our results also demonstrate this behavior in human brain (Fig. 2) and a phantom built specifically to demonstrate this behavior (Fig. 4), as well as in simulations using phantom data, high resolution *ex vivo* data from rat spinal cord, and human brain (Fig. 5). Findings by Billiet *et al*.^7^ indicated the kurtosis anisotropy to be unaffected by age related effects on brain microstructure. However, the precise definition of kurtosis anisotropy employed in that study was not reported, although it was possibly similar to the ref. 5 definition. That definition was compared to KFA in^11^ (where it was referred to as KA_σ_), where they were observed to offer markedly different contrast. However, if KFA is indeed insensitive to age related effects, it would be very important for the unbiased study of brain pathology in the elderly, where FA is strongly affected by age^30^ with an estimated decline of 3% per decade in the frontal lobe. Another potential application of KFA is as a supplement to brain fiber tractography, where its robustness to fiber crossings may provide information to alleviate the fiber crossing problem. Inclusion of non-Gaussian effects was shown to offer improved fiber direction profiles already in^13^, but so far no studies have explored the effect of including KFA in tractography.

While some of the above mentioned properties of KFA were already demonstrated in previous work, we confirmed them here using an extensive range of model systems. This serves both to supplement earlier work and as a basis for evaluation of the KFA proxy proposed in Eq. (4). The motivation for proposing the proxy is that (like conventional DKI in general) its evaluation may be too time consuming for the acute setting because its evaluation requires a large amount of data and non-trivial post-processing to reconstruct the full kurtosis tensor from which it is calculated. This makes KFA an unlikely choice for anisotropy estimation in situations where time is of essence such as the acute setting. The potential clinical utility of KFA may therefore go unexplored if not a rapid estimation scheme exists. One prominent example of this is acute stroke, where diagnosis may be improved by methods offering enhanced sensitivity to tissue changes but where imaging time is strongly limited due to patient concerns. Although the proposed KFA proxy does not directly correspond to true KFA obtained from W, it may still be useful in the acute setting where short acquisition and post-processing time may be more important than true KFA estimation as long as important aspects of KFA contrast is preserved by the proxy. Our investigation showed that the correlation between KFA and its proxy is excellent (>0.9) in both WM and GM under ideal conditions where SNR is high and many sampling directions are acquired (Fig. 6). Relaxing these conditions we found that SNR is the main determinant for proxy performance and that the proxy correlates well with KFA even for as few as nine sampling directions and an SNR of approximately 80 at b = 0. However, at typical clinical system SNR levels of ~40 at b = 0 the proxy’s correlation to true KFA was seen to be relatively poor in agreement with simulations. Interestingly, in rat we saw similar performance (linear correlation of approximately 0.5 between the proxy obtained with 1-9-9 and true KFA, Fig. 9A,B), although in this data set the SNR was higher (~75) at b = 0. This behavior is in agreement with simulations (Fig. 9C) indicating different SNR requirements. Further analysis revealed vastly different optimal b-values for the KFA proxy in fixed brain tissue and *in vivo*. This is not too surprising since fixation is known to alter tissue properties such as diffusivities and membrane permeabilities. Other causes of this behaviour may be differences in the size of structures in fixed rat brain and human brain. These effects all cause the tensors D and W that we probe to be quite different in the two cases. Furthermore, differences in encoding beyond b-value (combinations of gradient strengths and diffusion timings) employed on pre-clinical and clinical systems may contribute also as noted in connection with results in Fig. 9.

We note that the b-values (b1 = 1 ms/μm^2^, b2 = 2.4 ms/μm^2^) used in simulations for *in vivo* performance evaluation are optimized values, and that the same b-values are then used for *ex vivo* evaluation. The b-values are used because these are the values that would most likely be employed for 1-9-9 estimation *ex vivo* but here full estimation of D and W is feasible so the proxy is less valuable in the *ex vivo* case. While these b-values were also found to be optimal for 1-9-9 estimation of 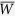 in fixed tissue in Ref. 20, they need not be optimal for FKA proxy estimation *ex vivo* where our analysis showed rather high optimal values of b_1_ = 1.6 ms/μm^2^ and b_2_ = 5.0 ms/μm^2^. These b-values are high but in fixed tissue diffusivities are much lower than *in vivo* so the b-values are still within the feasible range for DKI 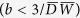. This means that if the proxy method is to be used in fixed tissue a 1-9-9-9 implementation would be needed with the nine directions acquired at one set of b-value pairs for estimation of 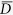, 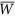, and FA and another b-value pair for proxy estimation of KFA.

In humans, the proxy method fares better and although the SNR requirement is high, estimation with nine directions is possible meaning that KFA may be properly evaluated as part of fast kurtosis methods such as^10^,^16^,^20^ which already provide fast and robust estimates of 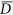, 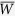, and FA. This is particularly encouraging because a recent study^31^ applying a fast kurtosis method in an animal model of acute stroke found it to be “capable of capturing heterogeneous diffusion and kurtosis lesions in acute ischemic stroke and thus is suitable for translational applications in the acute stroke clinical setting”. This is in agreement with previous studies employing traditional kurtosis methods in the assessment of stroke^17^,^32^,^33^,^34^. In combination with fast imaging strategies (e.g. multi-slice DWI imaging^35^), the 19 images required for fast estimation of 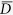, 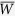, and FA as shown in^20^ may be obtainable with enough averaging to provide sufficiently high SNR to also allow a robust estimation of KFA by proxy all within an acquisition time suitable for the acute setting. An optimized protocol is the subject for future work.

Outside of clinical imaging, the principles of the protocol may also be of use e.g. in diffusion weighted spectroscopy where it is possible to probe the diffusion properties of compartment specific reporter molecules^36^. Such techniques might be another example where acquisition of a full diffusion kurtosis data set is not feasible but where a strategy providing four metrics from 19 high SNR spectra might be valuable.

## Conclusion

We explored the contrast provided by KFA in a number of model systems and in human brain. Phantom measurements and simulations were used to confirm the different behaviors of the DKI metrics in the presence of fiber distribution heterogeneity where FA fails as observed in human brain. KFA can therefore supplement FA in assessing tissue anisotropy and may therefore be useful for assessment or identification of complex fiber arrangements. A KFA proxy was suggested and a compact strategy for its estimation was evaluated. The proxy was found to correlate strongly with true KFA even for as few as nine sampling directions in high SNR conditions. In principle, this makes it possible to integrate estimation of the KFA proxy into the fast kurtosis scheme in^20^.

## Methods

All animal work was performed in accordance with relevant guidelines and regulations concerning animal experiments. All animal experimental protocols were approved by the Danish The Animal Experiments Inspectorate (Dyreforsøgstilsynet). Human data acquisition was performed in accordance with the Declaration of Helsinki. All human experimental protocols were approved by the local ethics committee for research (De videnskabsetiske Komitéer for Region Midtjylland). Informed consent was obtained from all human subjects (one) prior to scanning. Smoothing and spatial filtering was not applied in any of the data sets. Throughout, SNR was calculated as the average signal in a homogenous region in the object imaged divided by the standard deviation of the signal in a background region, corrected for Rayleigh distribution in a standard manner^37^. Unless otherwise stated reported SNR levels were evaluated at b = 0.

### MRI data obtained in fixed rat spinal cord

An adult male Wistar rat was euthanized and exsanguinated during intra-aortic perfusion fixation with isotonic saline containing heparin (10 IU/mL), followed by 4% paraformaldehyde in phosphate-buffered saline (PBS) (pH 7.4). A section of spinal cord including the cervical enlargement was then dissected out and stored in 4% PFA for weeks prior to imaging. The spinal cord segment was washed in PBS for 24 hours prior to MR scanning to improve signal by removal of excess fixative. For imaging, the tissue was placed in a 5 mm NMR tube. Imaging was performed on a Bruker Biospec 16.4T (Bruker Biospin, Germany) spectrometer equipped with microimaging gradients with a strength of 3T/m. Data was acquired using a 5 mm saddle coil. DWI data acquisition was performed using a standard DW spin echo sequence. A total of 17 b-values equally distributed from 0–15 ms/μm^2^ were acquired. At each b-value, data was acquired along 9 gradient directions, so that the gradient directions at non-zero b-values in combination form a 144 point spherical design^38^. Imaging parameters were: TE = 15.3 ms, TR = 2500 ms, diffusion timings δ/Δ = 2/8 ms, 3 averages. Acquisition time per b-value: 3 hrs 36 min. Twenty-five image slices were acquired at a resolution of 23 μm × 23 μm × 120 μm, matrix size 192 × 192.

### MRI data obtained in fixed rat brain

This specimen was obtained using the same fixation protocol as above. After perfusion fixation the brain was removed and immersion fixed in fresh 4% paraformaldehyde solution for weeks. Prior to imaging, the brain was washed in PBS for 24 hours to improve signal by removal of excess fixative. Data was acquired using a Bruker Biospec 9.4T (Bruker Biospin, Germany) MRI system equipped with a 15 mm quadrature coil. DWI data acquisition was performed using a standard DW spin echo sequence. A total of 15 b-values ranging from 0–3 ms/μm^2^ in steps of 0.2 ms/μm^2^ were acquired. At each b-value, data was acquired along 33 gradient directions. These directions were obtained by combination of a 3-dimensional 24-point spherical 7-design^38^ and the nine directions identified for fast estimation of mean kurtosis in^10^. Imaging parameters were: TE = 23.3 ms, TR = 4 s, diffusion timings δ/Δ = 4/14 ms, 2 averages. Fifteen image slices were acquired at a resolution of 100 μm × 100 μm × 500 μm, matrix size 128 × 128. SNR was approximately 75 at b = 0 evaluated using the mean signal across all tissue.

### MRI data collected in a physical phantom

Here, we wanted to construct a physical phantom with fiber bundles equally distributed along the 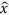, 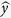, and 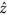 directions. This phantom was used to mimic one imaging voxel with complex fiber distribution while at the same time allowing us to resolve each fiber direction separately. For this, a phantom was built using fresh asparagus stems. Stems were cut into 8 mm long sections and placed inside a cubic plastic container in a 3 × 3 design (see Fig. 3A). The box was then placed in an in-house built sample holder made from PE foam allowing the sample to be held tightly in place inside the MR coil. Imaging was performed on a horizontal 9.4T Bruker Biospec system using a 40 mm quadrature coil. This coil is intended for mounting on an animal bed, but for these scans it was mounted in the magnet bore using a coil holder developed in-house. The scan protocol included an anatomical/structural scan and a DKI acquisition. The structural data was acquired with a FLASH sequence (TE = 5.4 ms, TR = 350 ms) in seven 1 mm thick slices with an in-plane resolution of 100 μm × 100 μm, matrix size 280 × 280. Diffusion data was acquired using a standard diffusion weighted spin echo sequence. Data was recorded in the same seven slice planes as the structural data but with a lower in-plane resolution of 427 μm × 427 μm, matrix size 64 × 64. Imaging parameters were TE = 70.6 ms, TR = 2700 ms, diffusion timings δ/Δ = 6/60 ms, 4 averages. Fifteen encoding directions were obtained at b-values of 0, 0.5, 1.0, 1.8, 2.5, 3.5 ms/μm^2^. The encoding directions were obtained from a 15 point spherical design^38^.

### Human MRI data

Human data was acquired in one normal volunteer using a Siemens Trio 3T equipped with a 32 channel head coil and a double spin echo DW EPI sequence. Motion of the subject’s head during acquisition was avoided by padding inside the coil. DWI data was recorded at b = 0 ms/μm^2^, and along 33 directions at b-values from 0.2–3 ms/μm^2^ in steps of 0.2 ms/μm^2^. The encoding scheme was constructed as a combination of a 24 point spherical design^38^ and the nine directions identified for rapid kurtosis estimation in^10^. CSF suppression (inversion recovery) was employed as recommended in^39^. Imaging parameters were TR = 7200 ms, TE = 116 ms, TI = 2100 ms, 19 consecutive slices were acquired at isotropic resolution of 2.5 mm, matrix size 96 × 96. SNR ~ 39 at b = 0 evaluated using the mean signal across all tissue types.

### Phantom data

The data from physical phantom was processed in two different ways. First, Eq. (1) was fitted to the data on a voxel-by-voxel basis producing maps of 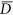, FA, 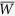, and KFA as before. Secondly, voxels outside the stems were masked out based on the 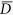 map from the voxel-wise fit (
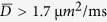
 excluded). Data from each diffusion encoding was then averaged across this mask and the averaged data was then fit to Eq. (1) again producing estimates of 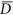, FA, 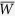, and KFA. Finally, parameter values from the voxel-wise fit were averaged across the same mask. One of the seven image planes acquired was omitted from the analysis due to partial volume effects at the top of the phantom.

### Data processing

All data sets were evaluated visually for quality (artifacts and subject movement). No artifacts (eddy currents or otherwise) were observed. Due to the padding around the subjects head, image registration was found to be unnecessary. All fits were performed using non-linear least squares optimization in Matlab^®^.

For DKI parameter estimation Eq. (1) was fitted to the data after normalization to the b = 0 image so that the fitted signal S(b)/S(b = 0) was in the range 0–1. Fitting was performed using non-linear least squares (lsqcurvefit with the ‘trust-region-reflective’ algorithm) as implemented in Matlab^®^. From this fit the full tensors D and W were obtained, and from these all relevant metrics were calculated as described above. This analysis was performed in all the data sets described above. When tissue segmentation is used FA ranges were used to segment the tissue into low anisotropy areas (dominantly GM) in the range 0.1 < FA < 0.3 and high anisotropy (WM, 0.6 < FA < 1) as in^20^.

### Simulating the effect of increasing tissue complexity on diffusion parameters

**Figure 10:**
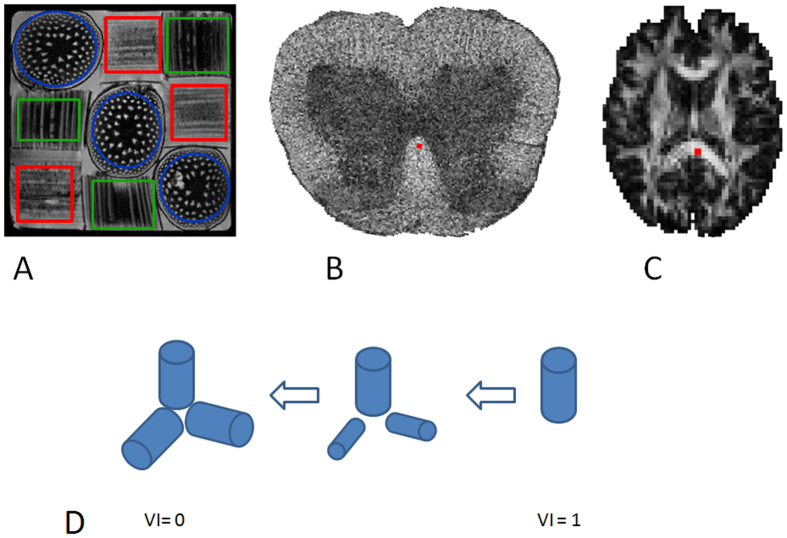
Panels (**A**–**C**) show the data basis for the simulations of the effect of increasing fiber orientation heterogeneity. Panel (**A**) shows the asparagus stalks in the phantom. ROIs were placed over each of the nine stalks and grouped by fiber orientation along x,y, and z with grouping indicated by the color coded region outlines. Panel (**B**,**C**) show FA maps obtained from microimaging of fixed rat spinal cord and from human brain respectively with the high anisotropy voxels that were used in the simulations outlined in red. Panel (**D**) illustrates the behavior of the volume fraction index (VI) with increasing fiber orientation heterogeneity.

A voxel of high FA was chosen in both the rat spinal cord data and the human data set (Fig. 10B,C in Methods). Based on position and FA value, these voxels are assumed to contain the signal from a bundle of very uniformly oriented fibers. A fit of a single fiber model^40^ in these voxels was then used to generate DWI signals from isolated fiber bundles oriented along 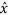, 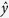, and 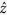. The signal was simulated along the 33 directions described above at each of the b-values 0.1–3.5 ms/μm^2^ in steps of 0.2 ms/μm^2^. For the case of the rat spinal cord data only b-values up 5.5 ms/μm^2^ were employed in the single fiber fit. The synthesized signals (termed *S*_*x*_, *S*_*y*_, and *S*_*z*_) were then mixed into a series of combined signals ranging from the signal from one single fiber direction (α = 0) to signal containing equal amounts (α = 1/3) of signal from the three fiber orientations. The mixed signals were produced in the interval α = 1/3 in steps of 0.03:


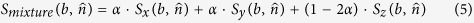


The DKI model (Eq.(1)) was then fitted to each of these mixture signals and the metrics 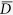, FA, 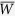 and KFA were calculated from each based on the tensors D and W as described in the theory section. Identical simulations were carried out with data from the physical phantom. Here, however, signals from fiber bundles along 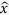, 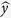, and 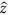 are directly available. Each signal component *S*_*x*_, *S*_*y*_, *S*_z_ was therefore obtained from regions of interest (ROIs) containing the stalks along each of the primary directions. Data from each bundle direction was normalized and then averaged across voxels. The regions/voxels used in the simulations are shown in Fig. 10A–C.

To construct a volume fraction index describing the fiber mixture we set up a diagonal tensor V with the volume fractions along the 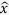, 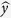, and 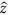 axis on the diagonal. The structure of the simulated tissue may then be described by the volume fraction index VI calculated in complete analogy to FA (Eq. (2)) except with the matrix V instead of D. A VI value of one would then describe a single fiber direction whereas equal amounts of fiber along the three axes would result in a VI of zero. The behavior of VI in our simulations is illustrated in Fig. 10D.

### Simulations for evaluation of the KFA proxy

#### SNR and encoding direction dependency

The fit to the human data described above was used for this part. From this fit W was obtained in each voxel and true KFA calculated using Eq. (3). This KFA map was used as reference in the simulations. Also using this fit, the DKI model (Eq. (1)) was used to simulate two shell acquisitions at b_1_ = 1 ms/μm^2^, and b_2_ = 2.5 ms/μm^2^. The b-values were chosen to follow the recommendations in^20^. In this manner, several two shell data sets were generated using sampling schemes with varying number of sampling directions: 3, 5, 9, 15, 25, 36, 46, 65, and 86, all sampling schemes generated using the tool available at http://www.emmanuelcaruyer.com/q-space-sampling.php. For each sampling scheme data sets were generated with varying levels of added Rician noise using 500 noise realizations for each SNR level. The KFA proxy was then estimated from all generated data sets and the correlations to the ‘true’ KFA from the ground fit set assessed and averaged over noise realizations. Simulations were performed based on both data from normal human brain (Fig. 6) and fixed rat brain (Fig. 9).

#### b-value dependency

For the b-value optimization the same fit to the human data was used as a basis for the simulations. A total of 125 random pixels across the brain were used for the simulation. Here the b-value combination was varied for a fixed number of sampling directions and a fixed SNR. For each b-value combination the linear correlation between the true KFA and the simulated KFA proxy over 500 noise realizations and the average linear correlation coefficient was then color coded onto the b_1_-b_2_-plane.

## Additional Information

**How to cite this article**: Hansen, B. and Jespersen, S. N. Kurtosis fractional anisotropy, its contrast and estimation by proxy. *Sci. Rep.* **6**, 23999; doi: 10.1038/srep23999 (2016).

## Figures and Tables

**Table 1:** The linear correlation coefficients between KFA and 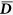, FA, and 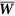 for the rat brain data in Fig. 1 and whole human brain (including the data in Fig. 2).

	Rat brain			Human brain		
	Linear correlationcoefficient 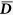 -KFA	Linear correlationcoefficient FA-KFA	Linear correlationcoefficient 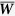 -KFA	Linear correlationcoefficient 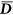 -KFA	Linear correlationcoefficient FA-KFA	Linear correlationcoefficient 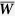 -KFA
All tissue	0.02	0.66	−0.02	−0.42	0.54	0.00
WM	0.05	0.48	−0.15	−0.15	0.23	0.08
GM	−0.09	0.22	−0.40	−0.39	0.17	−0.30
